# Unbearability of suffering at the end of life: the development of a new measuring device, the SOS-V

**DOI:** 10.1186/1472-684X-8-16

**Published:** 2009-11-03

**Authors:** Kees DM Ruijs, Bregje D Onwuteaka-Philipsen, Gerrit van der Wal, Ad JFM Kerkhof

**Affiliations:** 1 Primary Care Center De Greev, Utrecht, the Netherlands; 2 Department of Public and Occupational Health, EMGO Institute for Health and Care Research, Expertise Center for Palliative Care, VU University Medical Center, Amsterdam, the Netherlands; 3 Department of Clinical Psychology, EMGO + Institute for Health and Care Research, VU University, Amsterdam, the Netherlands

## Abstract

**Background:**

Unbearable suffering is an important issue in end-of-life decisions. However, there has been no systematic, prospective, patient-oriented research which has focused on unbearable suffering, nor is there a suitable measurement instrument. This article describes the methodological development of a quantitative instrument to measure the nature and intensity of unbearable suffering, practical aspects of its use in end-stage cancer patients in general practice, and studies content validity and psychometric properties.

**Methods:**

Recognizing the conceptual difference between unbearability of suffering and extent or intensity of suffering, we developed an instrument. The compilation of aspects considered to be of importance was based on a literature search. Psychometric properties were determined on results of the first interviews with 64 end-stage cancer patients that participated in a longitudinal study in the Netherlands.

**Results:**

The instrument measures five domains: medical signs and symptoms, loss of function, personal aspects, aspects of environment, and nature and prognosis of the disease. Sixty nine aspects were investigated, and an overall score was asked. In 64 end-stage cancer patients the instrument was used in total 153 times with an average interview time varying from 20-40 minutes. Cronbachs alpha's of the subscales were in majority above 0.7. The sum scores of (sub)scales were correlated strongly to overall measures on suffering.

**Conclusion:**

The SOS-V is an instrument for measuring the unbearability of suffering in end-stage cancer patients with good content validity and psychometric properties, which is feasible to be used in practice. This structured instrument makes it possible to identify and study unbearable suffering in a quantitative and patient-oriented way.

## Background

Suffering of patients is inseparably connected with the work of the medical profession. Serious disease can result in serious suffering. At some point, it is possible that patients may consider their suffering unbearable. Some of these patients may request euthanasia.

In the Netherlands, the discussion about euthanasia started in the early seventies, and unbearable suffering was the central theme [1]. In 2002, an act regulating euthanasia, in which unbearable suffering is one of the pivotal criteria for due care [2].

Since 1991, studies have been carried out in the Netherlands to achieve transparency in the frequencies of end-of-life decisions [1-5]. Given an estimated number of 8.400 explicit requests for euthanasia each year, at a total of about 140.000 deaths, patients consider their suffering unbearable in the last phase of life in at least 6% of all deaths [6]. The estimated frequency of euthanasia and physician-assisted suicide was 1,8 percent of all deaths in 2005. The majority (87 percent) of these deaths took place in primary care, and cancer was the most frequent diagnosis (84 percent) [2,6]. However, all these studies were physician-oriented.

The transparency about the nature and frequency of end-of-life decisions in medical practice in the Netherlands is not paralleled by a clear understanding of what patients consider unbearable suffering, since in nearly four decades the nature of unbearable suffering has not yet been studied through patient-oriented research [7]. Important reasons for studying unbearable suffering this way are the understanding of unbearable suffering, analysis of end-of-life decisions, and assessing and directing care needed.

We planned a longitudinal, prospective, quantitative patient-oriented study of the nature and intensity of unbearable suffering in end-stage cancer patients in primary care. In 2001, in preparation of our study, we performed a Medline literature search for patient-oriented studies focusing on unbearable suffering. Key words we used in different combinations were: unbearable suffering, suffering, quality of life, measure, cancer patients, palliative, general practice, primary care, euthanasia. We also studied a sample of convenience from the Dutch medical literature concerning unbearable suffering and end-of-life decisions. A compilation of over 200 articles related to the subject was studied. No patient-oriented research investigating unbearable suffering was found. Above that, no measurement instrument for unbearable suffering was found. At first glance, a quality-of-life instrument would appear an option. However years of clinical, and research experience within the research group showed a recurring phenomenon: in patients in whom no difference in quality of life was observed or expressed some would consider their suffering unbearable and ask for euthanasia, and others would not. In other words, the extent to which burdensome signs or symptoms are present does not necessarily parallel the experience of unbearability. This is what distinguishes unbearable suffering from health-related quality of life which is usually measured by assessing the extent to which signs or symptoms are present.

We decided to develop an instrument to measure the unbearability of suffering at the end of life. This article describes its development, giving insight in the content validity of the measurement instrument. Furthermore, we describe our experience with the practical use of the instrument in end-stage cancer patients and the psychometric properties of the instrument.

## Methods

For the systematic development of the instrument we defined unbearable suffering, decided whether or not non-disease-related aspects should be included, defined an integral framework of the main domains of suffering, implemented specific aspects of suffering in the framework, and developed a method of questioning patients about (un)bearability. The research group provided expertise in medicine (especially general practice and public health), psychology and health sciences.

### Definition

We defined unbearable suffering as a subjective experience of suffering that is so serious and uncontrollable that it overwhelms ones bearing capacity. This experience is not a steady state, but a state that fluctuates in intensity. This corresponds with the experience of physicians, who observe how a patient can feel unwell one day, and then better on another day. This can for instance depend on activities during the day, social contacts or treatment, or can have no apparent explanation.

### Framework of suffering and process of selection of aspects

The experience of suffering is a multidimensional entity with physical, psychological, social, existential and spiritual dimensions [8-10]. Thus, in the medical professional field, suffering will comprise disease-related and non-disease-related aspects and an integral instrument should account for both, resulting in a comprehensive measurement of suffering. A second reason to include non-disease-related aspects is the relationship between unbearable suffering and end-of-life decisions, which in it's essence are existential decisions [11]. In the literature we found two frameworks for the experience of suffering [12,13] with focus upon the domains of suffering as encountered in medical practice. We selected a framework with five domains: (I) medical signs and symptoms, (II) loss of function, (III) personal aspects, (IV) aspects of environment, (V) nature and prognosis of the disease [12]. Therefore, we named the instrument the State Of Suffering-Five (for five domains), in short: SOS-V 9 Additional file 1).

A compilation was made of aspects of suffering that were considered to be of importance in the studied literature, when focusing on end-stage cancer patients [1,4,7-9,11,14-33]. In a process of reflection and interaction the research group selected aspects and divided them over the five specific domains. Since this study originated in medical practice, emphasis was laid on the domain of medical signs and symptoms.

The items listed can predominantly be considered as causal indicators, which may cause unbearable suffering if present, while it is not necessary that people who suffer unbearably score high on these items. Therefore, in developing a valid instrument an important aim is that the list of items is comprehensive. This as opposed to selecting as few items as possible to validly measure a concept, as is done in items that can be considered effect indicators (e.g. depression screening scales) [34,35]. Focussing on comprehensiveness, we selected 69 aspects, while working towards an interview time of 30-45 minutes. Since a study among terminally ill cancer patients showed that for a majority of patients an interview with median duration of 83 minutes was acceptable [36], this could even leave time to address other topics in the interview.

### Measuring unbearable suffering

The next step was to formulate a question with which to measure unbearable suffering per aspect. Quality of life studies generally measure the extent or intensity of a symptom or complaint. This is important information, but as such it does not determine the unbearability of the suffering. A high score for an aspect of suffering might be unbearable for one person, but bearable for another person. Therefore, we concluded that two questions would be necessary: first asking about the intensity or extent to which an aspect was present, followed by a question on how unbearable it was.

### Eligibility criteria and other interview- and study information

Because of the target population of end-stage cancer patients, the mode of gathering information was chosen to be an interview. The patients were handed a card with the answering options to help choosing their answer. For this study, with the purpose of recording change in the last phase of life, the recall period of the instrument was 'the last two days'.

The interview was tested four times in patients with end-stage cancer who only had a few months to live, after which a few questions were reformulated. The resulting instrument was used in the actual study which was carried out in primary care in Utrecht, one of the four largest cities in the Netherlands. The study was approved by the ethical committee of VU University Medical Center. Patients were eligible if they had incurable cancer, an estimated life-expectancy of six months or less, were competent, had adequate command of the Dutch language, were expected to stay mostly at home, and had a general practitioner as the primary responsible physician. Sixty four patients were followed from inclusion until they deceased.

Forty five general practitioners invited eligible patients to participate in the study. The interviews took place in the patients home every two months, or sooner if the condition of the patient deteriorated (information provided by the general practitioner). In this paper, the data on the first interviews were used. In the first interview we used the SOS-V and several other instruments, among which the EORTC QLQ C30 [14], a health-related quality of life measurement for cancer patients. If, during the interview, the interviewer sensed that the general condition of the patient was too poor, the interview was cut short. Then preference was given to the SOS-V. The interview time was estimated approximately. At the end, the patient was asked how he or she had experienced the interview., This was not rated as a score, but was asked more openly, in order to end the interview on a more informal note.

### Translation of the instrument

The original language of the study was Dutch. Therefore, the validation of the instrument concerns the Dutch version. For the purpose of international publications, a back and forward translation into English took place. The instrument was translated to English by a native speaker, after which it was translated back into Dutch by a researcher not involved in this study. The few differences found were discussed between the two translators and one of the researchers (BOP) to decide on the appropriate English translation.

### Analysis of psychometric properties

Internal consistency was analysed with cronbachs alpha's for the 5 domains and for the total of aspects. This was done separately for the questions on the presence of the aspect and for the questions on the unbearabilty of these aspects. To examine the extent to which the conceptual difference between (a) the presence of aspects, and (b) the extent to which they lead to unbearable suffering actually exists in practice, scatter plots of presence (a) versus the sum cores of unbearability (b) were made for the sums cores per domain. Furthermore, the correlations between sum scores of the 5 domains and overall measures on extent of, unbearability of and hopelessness of suffering were calculated. Finally, the correlation between the sum scores of the sub domains and sum scores of the total SOS-V, and the sum score of the EORTC QLQ C-30 was calculated.

## Results

### The SOS-V

Table 1 shows the SOS-V, with the five domains of experience of suffering which we distinguished, the division of 69 aspects of suffering over these domains, and the additional questions. The domain "medical signs and symptoms" consists of most aspects: 37 aspects ordered following organ and functional systems.

**Table 1:** Domains and aspects of the SOS-V

**Domains**	**Aspects**
**I. Medical signs and symptoms**	**General**: 1: General discomfort (feeling miserable, feeling unwell) 2: Tired 3: Weakened 4: Not sleeping well 5: Pain 6: Loss of appetite 7: Thirst 8: Smelling unpleasant 9: Changed appearance **Psychological**: 10: Impaired clarity of thought 11: Loss of concentration 12: Memory loss 13: Feeling tense 14: Feeling depressed 15: Feeling anxious. **Respiratory tract**: 16: Shortness of breath 17: Coughing **Gastrointestinal tract and urinary tract**: 18: Swallowing and oesophageal passage obstructed for food 19: Swallowing and oesophageal passage obstructed for fluids 20: Nausea 21: Vomiting 22: Constipation 23: Diarrhoea 24: Intestinal cramps 25: Incontinence of urine 26: Incontinence of faeces 27: Hiccups **Skin**: 28: Pressure ulcers 29: Itch 30: Skin metastasis **Nervous and loco-motor system**: 31: Paralyzed limbs 32: Impaired co-ordination of movements 33: Incomprehensible speech 34: Impaired comprehension of speech 35: Dizziness 36: Impaired sight 37: Impaired hearing

**II. Loss of function**	38: Impaired working capacity 39: Impaired performance of routine daily activities 40: Impaired leisure activities 41: Help needed with housekeeping (shopping, cleaning the house) 42: Help needed with self-care (washing, dressing, eating, visit to the bathroom) 43: Bedridden 44: Restricted sexuality

**III. Personal aspects**	**Self-appraisal**: 45: Not satisfied with your own self (with who you are as a person) 46: Lived a life with little purpose 47: Experienced little success in life 48: Experienced little happiness with family, partner for life and/or friends 49: Trouble accepting present situation 50: Negative thoughts or worrying 51: Feelings of guilt 52: Feelings of worthlessness 53: Feelings of loneliness 54: Feelings of hopelessness 55: Feelings of not any longer being the same person 56: Feeling tired of life **Experience of loss of independence**: 57:Feeling dependant on others 58: Feeling loss of control over your own life 59: Feeling of being a nuisance to others **Experience of future perspective**: 60: Feeling of no longer being of importance to others in the remaining time 61: Feeling no longer able to do the things you consider important in the remaining time

**IV. Aspects of social environment**	**Relationship with family and friends**: 62:Feeling insufficiently supported by family, friends and those nearby 63: Feeling lonely because the most important people in your life are not there for you 64: Feelings of shame 65: Experiencing that those who are near by consider your suffering too severe **Communication**: 66: Unsatisfactory contact with family, friends and those who are near by **Aspects of care**: 67: Insufficient availability of care.

**V. Nature and prognosis of disease**	68: Fear of future suffering 69: Fear of not any longer having the strength to bear the suffering

**Missing aspects**	70: Mention any aspects missing, and score correspondingly

**Total score**	71: How severe is your suffering overall? 72: How unbearable is your suffering overall? 73: How hopeless is your suffering overall?

**Additional questions**	Four open ended questions (see appendix: the complete instrument)

Table 2 shows the two questions we used per aspect to measure the presence of the aspect, and the extent to which it was unbearable. Since unbearable suffering was defined as a condition that is present to a certain degree, it was possible to measure it in a range of scores. We constructed a five point scale with a parallel description for scoring. To keep the scoring simple we chose a uniform scale for both questions. This had consequences for the way in which the questions were formulated. The second question was not asked if the first score was 1. If a patient scored 4 or 5 for unbearability on an aspect, then in an open-ended question the patient was asked to specify the experience. To ensure comprehensiveness, after posing questions on the 69 aspects, the patient was asked to name any aspects of suffering that had not been mentioned. After that, there were three questions on the overall severity, unbearability, and hopelessness of the respondents' suffering. To analyse dimensions which determine the capacity to bear suffering the interview was ended with four open questions addressing the nature of one's capacity to bear suffering, the role of spirituality, the influence of previous experience of suffering caused by disease and unexpected positive consequences of one's disease. The complete instrument can be seen in the appendix.

**Table 2:** Questions about the unbearable suffering

	***For every aspect of the SOS-V the following two questions were asked:***
1	Do you feel .......(aspect)? Is there ....... (aspect)? *If the answer on question 1 indicated that the aspect was present (= all answers other than 'not at all') question 2 was posed:*
2	How unbearable was this? *If the answer on question 2 was 'seriously' or 'very seriously' more information on this was asked in an open-ended question*.

	Scale for question 1 and 2:
	not at all - slightly - moderately - seriously - very seriously (could hardly be worse)
	1 - 2 - 3 - 4 - 5

### Practical use of the instrument

Our data collection started May 2003, and the inclusion period lasted until April 2006. In the study group of 64 patients 175 interviews were held, 130 (76%) of which were complete. Four times the interview was impossible, because of the poor condition of the patient. The remaining 45 interviews were incomplete in the sense that not all the different sub-instruments had been administered; in 22 of these interviews the SOS-V was not completed. Thus the SOS-V was completed in 153 interviews (85%). Two interviews were interrupted because the patient was too tired; on these occasions the interview was completed a few days later. Completing the interview took approximately 60-75 minutes. In general, the completion of the SOS-V took 20-40 minutes. When it took longer, this was generally because the patients appreciated the opportunity to talk about things that they felt were important. They were given enough time to do so. As the experience of the interviewers increased, the interview time tended to decrease. In general, the interviews were experienced positively by the patients.

Eleven patients (17%) named 20 aspects in response to the question to name aspects of their suffering that were not among the 69 aspects. The analysis showed that seven aspects, that were all mentioned once, were really additional: cramps in the extremities, oedema of feet, tingling of feet after chemotherapy, poly urea, cold extremities, irritation and intolerance due to loss of naivety, and the legal bureaucracy involved in obtaining income when sick. The other thirteen aspects mentioned, were attributable to existing aspects of the SOS-V.

### Psychometric properties

Table 3 shows the average scores on presence and unbearability of the individual aspects for the first interviews. Most frequently the averages are between 1 and 2. This is related to the frequency an aspect is not present (and thus also not unbearable). Of the aspects 47 of 69 aspects is present in less than 50% of respondents. Table 4 shows that the cronbach's alpha for the subscales varied between 0.57 and 0.89, and that most subscales had a cronbach's alpha of over 0.7. This level can be seen as a level of sufficient internal consistency[37]. The internal consistency of the total SOS-V was substantial with cronbachs alpaha's of 0.90 for presence of aspects and 0.93 for unbearability of aspects. Furthermore, for all (sub)scales the cronbach's alpha's were higher for the questions on unbearability of aspects than for the questions on presence of aspects.

**Table 3:** Average scores for presence (a) and unbearability (b) of the individual SOS-V aspect in the first interviews (n = 64)*

**Aspect**	**a**.	**b**.	**Aspect**	**a**.	**b**.
*Domain I*			Domain II		
1 General discomfort	2.4	2.4	38 Impaired working capacity	1.7	1.6
2 Tired	3.2	2.7	39 Impaired in daily activities	3.2	3.2
3 Weakened	3.1	2.9	40 Impaired leisure activities	3.2	2.8
4 Not sleeping well	1.9	1.8	41 Needing help housekeeping	3.4	2.7
5 Pain	2.3	2.2	42 Needing help self-care	1.9	1.6
6 Loss of appetite	2.3	2.0	43 Bedridden	2.1	2.0
7 Thirst	2.1	1.5	44 Restricted sexuality	2.0	1.5
9 Smelling unpleasant	1.6	1.5	Domain III		
10 Impaired mental clarity	1.9	1.9	45 Not satisfied with own self	1.3	1.2
11 Concentration loss	2.1	1.9	46 Lived life with little purpose	1.3	1.1
12 Memory loss	2.0	1.7	47 Little success in life	1.2	1.2
13 Feeling tense	1.9	1.7	48 Little happiness with family	1.4	1.4
14 Feeling depressed	1.5	1.4	49 Trouble accepting situation	2.2	2.3
15 Feeling anxious	1.4	1.3	50 Negative thoughts or worrying	1.7	1.7
16 Shortness of breath	2.1	2.7	51 Feelings of guilt	1.2	1.2
17 Coughing	1.5	1.3	52 Feelings of worthlessness	1.4	1.4
18 Swallowing [..] food	1.6	1.6	53 Feelings of loneliness	1.5	1.3
19 Swallowing [..] fluid	1.3	1.4	54 Feelings of hopelessness	1.5	1.5
20 Nausea	1.5	1.6	55 Feeling no longer same person	1.5	1.4
21 Vomiting	1.4	1.6	56 Feeling tired of life	1.2	1.3
22 Constipation	1.6	1.5	57 Feeling dependent of others	2.8	2.6
23 Diarrhoea	1.2	1.2	58 Feeling loss of control life	1.6	1.7
24 Intestinal cramps	1.4	1.4	59 Feeling being a nuisance	1.7	1.7
25 Incontinence of urine	1.3	1.3	60 Feeling not important to others	1.3	1.3
26 Incontinence of faeces	1.1	1.1	61 Doing Important things not possible	2.6	2.6
27 Hiccups	1.3	1.4	Domain IV		
28 Pressure ulcers	1.1	1.1	62 Not supported sufficiently	1.1	1.1
29 Itch	1.4	1.3	63 Lonely/people not there for you	1.4	1.4
30 Skin metastasis	1.1	1.0	64 Feelings of shame	1.1	1.1
31 Paralyzed limbs	1.1	1.1	65 Close ones consider suffering severe	1.6	1.6
32 Impaired coordination	2.0	2.0	66 Unsatisfactory contact close ones	1.2	1.2
33 Incomprehensible speech	1.6	1.7	67 Insufficient availability of care	1.4	1.4
34 Impaired comprehension speech	1.1	1.1	Domain V		
35 Dizziness	1.5	1.5	68 Fear of future suffering	1.9	2.0
36 Impaired sight	1.7	1.6	69 No longer having strength to suffer	1.8	1.8
37 Impaired hearing	1.8	1.7			

* Average of score between 1 (not at all) and 5 (very seriously); if an aspect was not at all present (a), it was scored also as not at all unbearable (b). Missing observations between 6 and 9

**Table 4:** Internal consistency of the SOS-V subscale and total used at the first interviews with patients in general practice with incurable cancer with a life-expectancy of 6 months or less (cronbach's α, n = 64)*

**(sub)scale**	**Number of items**	**a. Aspect present?**	**b. Suffering Unbearable?**
Domain I: Signs and symptoms	37	0.79	0.87
Domain II: Loss of functions	7	0.70	0.72
Domain III: Personal aspects	17	0.82	0.89
Domain IV: Aspects of social environment	6	0.57	0.62
Domain V: Nature and prognosis of disease	2	0.64	0.72
			

Total SOS-V	69	0.90	0.93

* between 6 and 8 missing observations per (sub)scale

**Figure 1:**
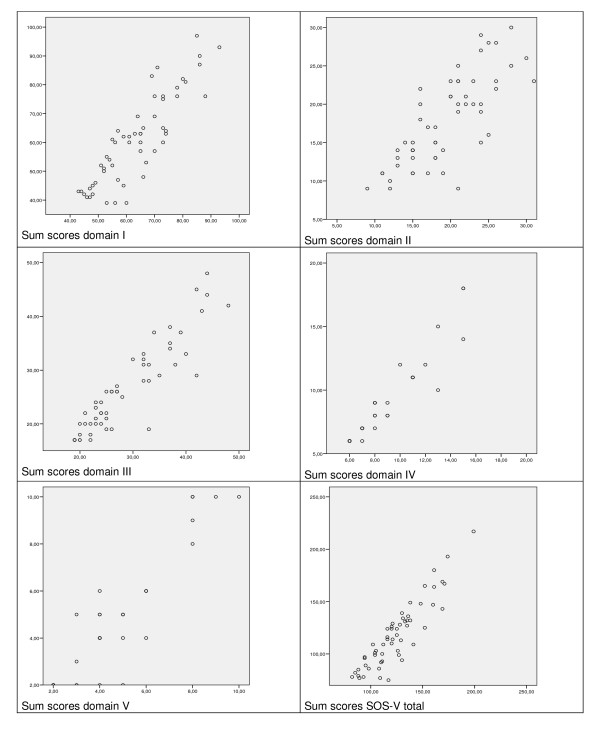
**Scatter plots of the sum scores on 'aspect present?' (x-axis) versus 'aspect unbearable?' (y-axis) for sub scales and total of the SOS-V**.

Figure 1 shows that for all (sub)scales the answers for presence were not identical to the answers for unbearability. However, they were strongly correlated, with correlations from 0,77 (for domain II) to 0.91 (for domain IV). For all (sub)scales, in the majority of cases the sum score for presence was higher than the sum score for unbearability.

Table 5 shows the correlations of sum scores of (sub)scales with the three overall questions on suffering. All but two correlations (between aspects of social environment and overall unbearability of suffering, and between nature and prognosis of disease and overall hopelessness of suffering) between subscales and overall measures were significant. In general, the (sub) scales' questions on extent of suffering were somewhat higher correlated to the overall extent of suffering than the overall unbearability of suffering; e.g. 0.72 versus 0.66 for the total scale. Similarly, the (sub) scales' questions on unbearability of suffering were somewhat higher correlated with the overall unbearability than with the overall extent of suffering. Domain II and IV were less correlated to the overall measures of suffering, compared to the other domains and the total SOS-V. The correlations of the (sub)scales with overall measure of hopelessness of suffering were generally somewhat lower than with the other two overall measures. An exception was domain IV for which the correlations were highest with overall hopelessness of suffering.

**Table 5:** Correlations between sum scores of SOS-V (sub)scales with patient with patient's general feeling of suffering and sum score of the QLQ C-30; first interviews with patients in general practice with incurable cancer with a life-expectancy of 6 months or less (Spearman R, n = 64)*

	**Overall Questions on suffering of the SOS-V**						**QLQ C-30**	
	**Extent of suffering**		**Unbearability of suffering**		**Hopelessness of suffering**		**Sum Score**	
**SOS-V** **(sub)scale**	**a. Aspect present?**	**b**. **Suffering Unbearable?**	**a. Aspect present?**	**b**. **Suffering Unbearable?**	**a. Aspect present?**	**b**. **Suffering Unbearable?**	**a**. **Aspect present?**	**b**. **Suffering Unbearable?**

Domain I: Signs and symptoms	0.71^‡^	0.70^‡^	0.51^‡^	0.42^‡^	0.64^‡^	0.74^‡^	0.71^‡^	0.61^‡^
Domain II: Loss of functions	0.33^†^	0.43^‡^	0.34^†^	0.41^‡^	0.28^†^	0.52^‡^	0.64^‡^	0.54^‡^
Domain III: Personal aspects	0.64^‡^	0.62^‡^	0.53^‡^	0.58^‡^	0.64^‡^	0.70^‡^	0.36^‡^	0.38^†^
Domain IV: Aspects of social environment	0.35^‡^	0.30^†^	0.46^‡^	0.43^‡^	0.29^†^	0.24	0.17	0.15
Domain V: Nature and prognosis of disease	0.56^‡^	0.57^‡^	0.33^†^	0.27	0.70^‡^	0.73^‡^	0.23	0.30^†^
								

Total SOS-V	0.72^‡^	0.69^‡^	0.61^‡^	0.55^‡^	0.66^‡^	0.75^‡^	0.66^‡^	0.58^‡^

* between 8 and 15 missing observations per correlation

^† ^significant p-value< 0.05

^‡ ^significant p-value< 0.01

While the correlations between the total SOS-V scores and the EORTC QLQ C30 were high (0.66 for aspect present and 0.58 for aspect unbearable), the correlations differed substantially per sub domain of the SOS-V: they were relatively high for the first and second domain and lower for the other domains, especially for domain IV. (Table 5)

## Discussion

Unbearable suffering may be feared or experienced by patients with an incurable disease, but it has not yet been the focus of patient-oriented studies. We carried out a prospective, patient-oriented study to investigate the nature and intensity of unbearable suffering, for which it was necessary to develop a measurement instrument. Based on a systematic study of the literature, we developed a quantitative measurement instrument. We hypothesize that unbearable suffering occurs when the suffering exceeds the bearing capacity of the individual. For some persons this may occur at the level of individual dimensions. For other persons unbearable suffering may occur when the sum of suffering of diverse dimensions exceeds the bearing capacity. The approach of a framework of suffering based on diverse domains may define a complementary pathway of palliative care, in which relieving measures of care in one domain may result in increased bearing capacity 'available' to bear suffering in another domain. This mechanism may also account for aspects of suffering within one domain.

Content validity refers to the extent to which the concepts of interest are comprehensively represented by the items of an instrument. Important in judging the content validity is whether the measurement aim, target population, and underlying concepts are taken into consideration appropriately in developing the instrument [37]. The above shows that this has been done in developing the SOS-V. Furthermore, in selecting items for the SOS-V we used literature, expert opinions and the target population. Although the pilot study was limited to a few patients of the target population, the use of the instrument in the study showed that most patients did not add extra items. This suggests that among the target population the item list is generally comprehensive. This opportunity to add relevant items is important, as suffering is a subjective experience. It is especially important when the instrument is used in other populations than the population for which it was developed. For instance, in the Netherlands, virtually all people have health insurance. Therefore, the financial impact of a life-limiting illness might be a more important aspect in countries with lower coverage of health insurance. Similarly, it is possible that in a secularized country like the Netherlands spiritual issues are different than in other countries.

The comparison with the overall scores on suffering suggests that the SOS-V actually measures suffering. The added value of the use of the SOS-V compared to overall scores is that it gives insight in the sources of suffering. The internal consistency of the instrument is acceptable, except may be for domain IV and V. However, it is debatable how necessary internal consistency is in an instrument of which the items are mainly causal indicators [35,36]. A limitation of the study is that it was not feasible to study test-retest reliability. In studies in patients in the last phase of life the retest should take place within less than a week of the first interview [37], and we assessed that this would limit the willingness to participate in the prospective part of the study with interviews every 2 months (a period too long for test-retest).

In the development of the instrument, the conceptual difference between the presence of an aspect and it's unbearability was an important starting point. The results show that there is indeed a difference between presence of aspects and unbearability. However, the difference is not as large as we expected. In interpreting the correlations between presence and unbearability, it should be understood that if an aspect was not present, by definition unbearability was also not present. This contributed to the high correlations, especially when many aspects were not present. In light of the conceptual difference between presence and unbearability, and the differences found between these (however small), we think it is important that the SOS-V consists of both questions. However, it could be debated that depending on the purpose of use of the SOS-V, and the need for briefness in the data collection one could opt for only one of the questions (either on presence or on unbearability).

The relatively high correlations of the SOS-V scores with the total score on the EORTC QLQ C30 are an indication for criterion validity of the instrument. At the same time, the fact that this high correlation is especially to be contributed to the first two domains -- 'medical signs and symptoms' and 'loss of function' -- confirms the difference between health-related quality of life and suffering. The SOS-V gives more information on the less medical, more psychosocial aspects of suffering. That this is relevant is indicated by the significant correlations between the sub domains III to V and the overall measures of suffering.

In our study of 64 end-stage cancer patients, 175 interviews were carried out. The SOS-V was completed 153 times, which demonstrates the feasibility of the instrument in practice. The interview time for the SOS-V varied between 20-40 minutes; the duration of the interviews was influenced by open-ended questions asking for more specific information if the scores were high, and also by the way in which the patients appreciated the opportunity to talk about their disease and its impact on their lives. For some patients the interview time was too long. After completion of other parts of the interview 22 times patients were too tired for the SOS-V, and four times the interview was impossible because of the poor condition of the patient.

## Conclusion

We conclude that with the development of the SOS-V we developed an instrument for measuring the unbearability of suffering in end-stage cancer patients with a good content validity and psychometric properties, which is feasible to be used in practice. This quantitative instrument to measure the unbearability of suffering offers an important step to better understanding the phenomenon of unbearable suffering through gaining information directly from patients. It also will make it possible to study whether palliative care interventions influence the experienced unbearability of suffering. Of course, when used in other countries, in other languages, or in other patient groups, attention for validation of the instrument is again important.

## Competing interests

The authors declare that they have no competing interests.

## Authors' contributions

All authors contributed to the design of the study and the development of the SOS-V. BOP is responsible for acquisition of funding. KR participated in data collection and drafted the manuscript. AK, GvdW, BOP have been in revising the manuscript for important intellectual content. All authors read and approved of the final manuscript.

## Pre-publication history

The pre-publication history for this paper can be accessed here:



## Supplementary Material

Additional file 1 **SOS-V**. the complete SOS-V measuring instrument. Click here for file
