# Supplementary material for: Unbearability of suffering at the end of life: the development of a new measuring device, the SOS-V
Source: BMC Palliat Care. 2009 Nov 3;8:16. doi: 10.1186/1472-684X-8-16 (PMC2777135; doi:10.1186/1472-684X-8-16)
Supplement: Additional file 1 — SOS-V. the complete SOS-V measuring instrument. [file 1472-684X-8-16-S1.pdf]

Appendix: State of Suffering Five (SOS-5): the complete instrument

A SOS-FIVE:INSTRUCTIONS FOR THE INTERVIEWER

The SOS-FIVE is a structured, quantitative interview to measure suffering and unbearability, with the possibility of supplementary qualitative questions. Scores are given per aspect of suffering for two categories: presence and unbearability; the scores for both questions are on a 5-point scale with a parallel description .The question about unbearability only follows if the score on the first question is two or higher. In case of a certain question not being applicable for a certain patient there exists an extra non-numerical scoring-opportunity X; this extra scoring possibility should be named along with the concerning question.

In case of a score 4 or 5 for the category unbearability the interviewer continues with a supplementary open type of question to clarify what the patient precisely considers unbearable. The question is directed at a short clarifying answer; the purpose is not to question very extensive, for the interview than may last too long and burden the patient; the essence of the answers and literary quotes are immediately written down. The interview ends with a few open type questions about background circumstances.

The scoring of the instrument is through personal interview; the questions are about ***the last two days****.*

The SOS-FIVE is a structured interview, in which the sequence of the questions is followed. If the patient earlier in the interview puts forward an aspect which is applicable further on in the interview, than the interviewer informs the patient the theme follows later in the interview. If considered opportune, one may also first continue with the aspect the patients puts forward and then turn back to the normal sequence of the interview.

RESPONSE SCORES

	**Not at all**	**Slightly**	**Modera-tely**	**Seriously**	**Very seriously#**
**1- ASPECT PRESENT?**	1	**2**	**3**	**4**	**5**
**2- IS THIS UNBEARABLE?**	1	2	3	**4***	**5***

-could hardly be worse
*-continue with open questions

B- INTERVIEW TECHNIQUE AND INSTRUCTIONS FOR THE PATIENT

INTERVIEW TECHNIQUE

1- ASPECT PRESENT?

Do you have …..?	(e.g. pain)
How ….. are you?	(e.g. tired)
In how far are …. ?	(e.g. not sleeping well)

At the moment the patient understands the system: only name the aspect.

2- IS THIS UNBEARABLE FOR YOU?

If score 4 or 5:	What is so unbearable??
If necessary continue:	And what do you find the most difficult?
	And what do you find the worst?
	About what are you most irritated?
	About what are you the most depressed?

INSTRUCTIONS FOR THE PATIENT::

Give the scoring-aid to the patient. Explain the scoring system with the help of the example on the scoring aid. Explain the scores apply for all questions and that a score "very seriously" means it hardly could be worse. Explain the questions consist of two parts. The first question is in how far a complaint or situation is present. The second question is in how far this complaint or situation is unbearable. Explain that if a high score (4 of 5) is given for the second question, there will be a successive question for a personal explanation. Give the instruction not to think too long about the score, but to react upon first impression. Give the instruction that at the end of the interview there will follow a few short questions informing about one's personal background.

DISCUSS ALL QUESTIONS ARE ABOUT THE LAST TWO DAYS.

C- SOS-5: QUESTIONS

	Not at all	Slightly	Mode-rately	Seriously	Very seriously	
**1- ASPECT PRESENT?**	1	**2**	**3**	**4**	**5**	
**2- IS THIS UNBEARABLE?**	1	2	3	**4***	**5***	**(*:continue with open questions)**

DOMAIN: MEDICAL SIGNS AND SYMPTOMS

A *General*	Aspect present?					Is this unbearable?				
1- General discomfort? (feeling miserable, feeling unwell)	1	**2**	**3**	**4**	**5**	1	2	3	**4**	**5**
2- Tired?	1	**2**	**3**	**4**	**5**	1	2	3	**4**	**5**
3- Weakened?	1	**2**	**3**	**4**	**5**	1	2	3	**4**	**5**
4- Not sleeping well?	1	**2**	**3**	**4**	**5**	1	2	3	**4**	**5**
5- Pain?	1	**2**	**3**	**4**	**5**	1	2	3	**4**	**5**
6- Loss of appetite?	1	**2**	**3**	**4**	**5**	1	2	3	**4**	**5**
7- Thirst?	1	**2**	**3**	**4**	**5**	1	2	3	**4**	**5**
8- Smelling unpleasant?	1	**2**	**3**	**4**	**5**	1	2	3	**4**	**5**
9- Changed appearance?	1	**2**	**3**	**4**	**5**	1	2	3	**4**	**5**

Space to write the given answers in case of additional open questioning	
Number of question	Answer

All questions are about the last two days

	**Not at all**	**Slightly**	**Mode-rately**	**Seriously**	**Very seriously**	
**1- ASPECT PRESENT?**	1	**2**	**3**	**4**	**5**	
**2- IS THIS UNBEARABLE?**	1	2	3	**4***	**5***	**(*: continue with open questions)**

B *Psychological*	Aspect present?					Is this unbearable?				
10- Impaired clarity of thought?	1	**2**	**3**	**4**	**5**	1	2	3	**4**	**5**
11- Loss of concentration?	1	**2**	**3**	**4**	**5**	1	2	3	**4**	**5**
12- Memory loss?	1	**2**	**3**	**4**	**5**	1	2	3	**4**	**5**
13- Feeling tense?	1	**2**	**3**	**4**	**5**	1	2	3	**4**	**5**
14- Feeling depressed?	1	**2**	**3**	**4**	**5**	1	2	3	**4**	**5**
15- Feeling anxious?	1	**2**	**3**	**4**	**5**	1	2	3	**4**	**5**

Space to write the given answers in case of additional open questioning	
Number of question	Answer

C *Respiratory tract*	Aspect present?					Is this unbearable?				
16- Shortness of breath?	1	**2**	**3**	**4**	**5**	1	2	3	**4**	**5**
17- Coughing?	1	**2**	**3**	**4**	**5**	1	2	3	**4**	**5**

Space to write the given answers in case of additional open questioning	
Number of question	Answer

All questions are about the last two days

	Not at all	Slightly	Mode-rately	Seriously	Very seriously	
**1- ASPECT PRESENT?**	1	**2**	**3**	**4**	**5**	
**2- IS THIS UNBEARABLE?**	1	2	3	**4***	**5***	**(*:continue with open questions)**

D *Gastrointestinal tract and urinary tract*	Aspect present?					Is this unbearable?				
18- Swallowing and/or oesophageal passage obstructed for food ?	1	**2**	**3**	**4**	**5**	1	2	3	**4**	**5**
19- Swallowing and/or oesophageal passage obstructed for fluid?	1	**2**	**3**	**4**	**5**	1	2	3	**4**	**5**
20- Nausea?	1	**2**	**3**	**4**	**5**	1	2	3	**4**	**5**
21- Vomiting?	1	**2**	**3**	**4**	**5**	1	2	3	**4**	**5**
22- Constipation?	1	**2**	**3**	**4**	**5**	1	2	3	**4**	**5**
23- Diarrhea?	1	**2**	**3**	**4**	**5**	1	2	3	**4**	**5**
24- Intestinal cramps?	1	**2**	**3**	**4**	**5**	1	2	3	**4**	**5**
25- Incontinence of urine?	1	**2**	**3**	**4**	**5**	1	2	3	**4**	**5**
26- Incontinence of faeces?	1	**2**	**3**	**4**	**5**	1	2	3	**4**	**5**
27- Hiccups?	1	**2**	**3**	**4**	**5**	1	2	3	**4**	**5**

Space to write the given answers in case of additional open questioning	
Number of question	Answer

All questions are about the last two days

	Not at all	Slightly	Mode-rately	Seriously	Very seriously	
**1- ASPECT PRESENT?**	1	**2**	**3**	**4**	**5**	
**2- IS THIS UNBEARABLE?**	1	2	3	**4***	**5***	**(*:continue with open questions)**

E *Skin*	Aspect present?					Is this unbearable?				
28- Pressure ulcers?	1	**2**	**3**	**4**	**5**	1	2	3	**4**	**5**
29- Itch?	1	**2**	**3**	**4**	**5**	1	2	3	**4**	**5**
30- Skin metastasis?	1	**2**	**3**	**4**	**5**	1	2	3	**4**	**5**

Space to write the given answers in case of additional open questioning	
Number of question	Answer

F Nervous and loco-motor system	Aspect present?					Is this unbearable?				
31- Paralyzed limbs?	1	**2**	**3**	**4**	**5**	1	2	3	**4**	**5**
32- Impaired co-ordination of movements?	1	**2**	**3**	**4**	**5**	1	2	3	**4**	**5**
33- Incomprehensible speech?	1	**2**	**3**	**4**	**5**	1	2	3	**4**	**5**
34- Inpaired comprehension of speech?	1	**2**	**3**	**4**	**5**	1	2	3	**4**	**5**
35- Dizziness?	1	**2**	**3**	**4**	**5**	1	2	3	**4**	**5**
36- Impaired sight?	1	**2**	**3**	**4**	**5**	1	2	3	**4**	**5**
37- Impaired hearing?	1	**2**	**3**	**4**	**5**	1	2	3	**4**	**5**

Space to write the given answers in case of additional open questioning	
Number of question	Answer

All questions are about the last two days

	Not at all	Slightly	Mode-rately	Seriously	Very seriously	
**1- ASPECT PRESENT?**	1	**2**	**3**	**4**	**5**	
**2- IS THIS UNBEARABLE?**	1	2	3	**4***	**5***	**(*:continue with open questions)**

DOMAIN: LOSS OF FUNCTION

Instruction for the patient: some of the following questions possibly do not apply for you; please name so if present?

Interviewer: score X if the question does not apply

		Aspect present?						Is this unbearable?				
38-	Impaired working capacity?	X	1	**2**	**3**	**4**	**5**	1	2	3	**4**	**5**
39-	Impaired performance of routine daily activities?	X	1	**2**	**3**	**4**	**5**	1	2	3	**4**	**5**
40-	Impaired leisure activities?	X	1	**2**	**3**	**4**	**5**	1	2	3	**4**	**5**
41-	Help needed with house-keeping? (shopping, cleaning the house)	X	1	**2**	**3**	**4**	**5**	1	2	3	**4**	**5**
42-	Help needed with self-care? (washing, dressing,eating,visit to the bathroom)	X	1	**2**	**3**	**4**	**5**	1	2	3	**4**	**5**
43-	Bedridden?	X	1	**2**	**3**	**4**	**5**	1	2	3	**4**	**5**
44-	Restricted sexuality?	X	1	**2**	**3**	**4**	**5**	1	2	3	**4**	**5**

Space to write the given answers in case of additional open questioning	
Number of question	Answer

All questions are about the last two days

	Not at all	Slightly	Mode-rately	Seriously	Very seriously	
**1- ASPECT PRESENT?**	1	**2**	**3**	**4**	**5**	
**2- IS THIS UNBEARABLE?**	1	2	3	**4***	**5***	**(*:continue with open questions)**

DOMAIN: PERSONAL ASPECTS

		Aspect present?					Is this unbearable?				
A	*Self-appraisal*										
45-	Not satisfied with your own self? (with who you are as a person)	1	**2**	**3**	**4**	**5**	1	2	3	**4**	**5**
46-	Lived a life with little purpose?	1	**2**	**3**	**4**	**5**	1	2	3	**4**	**5**
47-	Experienced little success in life?	1	**2**	**3**	**4**	**5**	1	2	3	**4**	**5**
48-	Experienced little happiness with family, partner for life and/or friends?	1	**2**	**3**	**4**	**5**	1	2	3	**4**	**5**
49-	Trouble accepting present situation?	1	**2**	**3**	**4**	**5**	1	2	3	**4**	**5**
50-	Negative thoughts or worrying?	1	**2**	**3**	**4**	**5**	1	2	3	**4**	**5**
51-	Feelings of guilt?	1	**2**	**3**	**4**	**5**	1	2	3	**4**	**5**
52-	Feelings of worthlessness?	1	**2**	**3**	**4**	**5**	1	2	3	**4**	**5**
53-	Feelings of loneliness?	1	**2**	**3**	**4**	**5**	1	2	3	**4**	**5**
54-	Feelings of hopelessness?	1	**2**	**3**	**4**	**5**	1	2	3	**4**	**5**
55-	Feelings of not any longer being the same person?	1	**2**	**3**	**4**	**5**	1	2	3	**4**	**5**
56-	Feeling tired of life?	1	**2**	**3**	**4**	**5**	1	2	3	**4**	**5**

Space to write the given answers in case of additional open questioning	
Number of question	Answer

All questions are about the last two days

	Not at all	Slightly	Mode-rately	Seriously	Very seriously	
**1- ASPECT PRESENT?**	1	**2**	**3**	**4**	**5**	
**2- IS THIS UNBEARABLE?**	1	2	3	**4***	**5***	**(*:continue with open questions)**

B	*Experience of loss of independence*	Aspect present?					Is this unbearable?				
	57- Feeling dependant on others?	1	**2**	**3**	**4**	**5**	1	2	3	**4**	**5**
	58- Feeling loss of control over your own life?	1	**2**	**3**	**4**	**5**	1	2	3	**4**	**5**
	59- Feeling of being a nuisance to others?	1	**2**	**3**	**4**	**5**	1	2	3	**4**	**5**

Space to write the given answers in case of additional open questioning	
Number of question	Answer

C	*Experience of future perspective*	Aspect present?					Is this unbearable?				
	60- Feeling of no longer being of importance to others in the remaining time?	1	**2**	**3**	**4**	**5**	1	2	3	**4**	**5**
	61- Feeling no longer able to do the things you consider important in the remaining time?	1	**2**	**3**	**4**	**5**	1	2	3	**4**	**5**

Space to write the given answers in case of additional open questioning	
Number of question	Answer

All questions are about the last two days

		Not at all	Slightly	Mode-rately	Seriously	Very seriously	
**1-**	**ASPECT PRESENT?**	1	**2** →	**3** →	**4** →	**5**	
**2-**	**IS THIS UNBEARABLE?**	1	2	3	**4*** →	**5***	**(*:continue with open questions)**

DOMAIN: ASPECTS OF ENVIRONMENT

		Aspect present?					Is this unbearable?				
A	*Relationship with family and friends*										
	62- Feeling insufficiently supported by family, friends and those near by?	1	**2**	**3**	**4**	**5**	1	2	3	**4**	**5**
	63- Feeling lonely because the most important people in your life are not there for you?	1	**2**	**3**	**4**	**5**	1	2	3	**4**	**5**
	64- Feelings of shame?	1	**2**	**3**	**4**	**5**	1	2	3	**4**	**5**
	65- Experience that those who are near by consider your suffering too severe?	1	**2**	**3**	**4**	**5**	1	2	3	**4**	**5**
B	*Communication*										
	66- Unsatisfactory contact with family, friends and those who are near by?	1	**2**	**3**	**4**	**5**	1	2	3	**4**	**5**
C	*Aspects of care*										
	67- Insufficient availability of care?	1	**2**	**3**	**4**	**5**	1	2	3	**4**	**5**

Space to write the given answers in case of additional open questioning	
Number of question	Answer

All questions are about the last two days

	Not at all	Slightly	Mode-rately	Seriously	Very seriously	
**1- ASPECT PRESENT?**	1	**2** →	**3**	**4**	**5**	
**2- IS THIS UNBEARABLE?**	1	2	3	**4*** →	**5***	**(*:continue with open questions)**

DOMAIN: NATURE AND PROGNOSIS OF DISEASE

	Aspect present?					Is this unbearable?				
68- Fear of future suffering?	1	**2**	**3**	**4**	**5**	1	2	3	**4**	**5**
69- Fear of not any longer having the strength to bear the suffering?	1	**2**	**3**	**4**	**5**	1	2	3	**4**	**5**

Space to write the given answers in case of additional open questioning	
Number of question	Answer

MISSING ASPECTS

Mention any aspects missing, and score correspondingly?

	Aspect present?					Is this unbearable?				
70- ………………………………………………………	1	**2**	**3**	**4**	**5**	1	2	3	**4**	**5**
71- ………………………………………………………	1	**2**	**3**	**4**	**5**	1	2	3	**4**	**5**
72- ………………………………………………………	1	**2**	**3**	**4**	**5**	1	2	3	**4**	**5**
73- ………………………………………………………	1	**2**	**3**	**4**	**5**	1	2	3	**4**	**5**

Remarks

All questions are about the last two days

TOTAL SCORE

Instruction for the patient: the following three questions are about **your situation as a whole**.
Interviewer: ask all three questions (indifferent of score)

	**Not at all**	**Slightly**	**Mode-rately**	**Seriously**	**Very seriously**	
1- How severe is your suffering overall?	1	2	3	4	5	
2- How unbearable is your suffering overall?	1	2	3	4*	5*	
3- How hopeless is your suffering overall?	1	2	3	4*	5*	(*: continue with open questions)

Answers in case of additional open ended questions

All questions are about the last two days

Final questions, open type (only the first interview):
1-We have talked about the suffering you experience. What makes it possible to bear the suffering?
2-Do you find relief in faith or conviction about life in your present situation?
3-Have you experienced serious suffering of somebody else due to disease in the past? What has been the influence upon you?
4-Did your disease by any chance also had positive consequences upon your life? Which were these consequences and why were they positive?
